# Fermentation Characterization of Chinese Yam Polysaccharide and Its Effects on the Gut Microbiota of Rats

**DOI:** 10.1155/2009/598152

**Published:** 2009-08-25

**Authors:** X. F. Kong, Y. Z. Zhang, X. Wu, Y. L. Yin, Z. L. Tan, Y. Feng, F. Y. Yan, M. J. Bo, R. L. Huang, T. J. Li

**Affiliations:** ^1^Key Laboratory of Agro-Ecological Processes in Subtropical Region, Institute of Subtropical Agriculture, The Chinese Academy of Sciences, Changsha, Hunan 410125, China; ^2^Graduate School of the Chinese Academy of Sciences, The Chinese Academy of Sciences, Beijing 100039, China

## Abstract

Rat was used to characterize Chinese Yam polysaccharides (CYPs). In *Exp. 1*, maximum volume and rate of gas production in CYP 3-supplemented group were higher than other CYP-supplemented groups and control group, while pH values and NH3 contents in CYP 2-, CYP 3-, and CYP 4-supplemented groups were lower than control group. Contents of acetate, propionate and butyrate increased by supplementing CYP 3 or CYP 4 compared to other groups, except for glucose-supplemented group. Contents of isobutyrate for CYPs groups decreased compared to control group. CYP 3 enhanced beneficial gut microbiota, but suppressed bacterial pathogens. In *Exp. 2*, contents of acetate and butyrate in cecal digesta of rats fed 0.25 or 0.5 g/kg CYP 3 were higher than other groups on day 7. pH values in 0.25 and 0.5 g/kg groups were lower than 1.0 g/kg group. Contents of acetate in 0.25 and 0.5 g/kg groups were greater than other 2 groups on day 21. Gut microflora in CYP 3-supplemented rats had greater diversity than non-supplemented rats. CYP 3 enriched beneficial gut microbiota, but suppressed bacterial pathogens in rat cecum. These findings suggested that CYP 3 is a good source of carbon and energy, and may improve bacterial community diversity and modulate short-chain fatty acid production in hindgut of rats.

## 1. Introduction

The main function of the gastrointestinal system is to assimilate nutrients from the external environment into the animal's internal environment, where they can be used for tissue growth and repair [1, 2]. The lumen of the large intestine contains billions of microorganisms. These microorganisms use the food residues that are discharged from the small intestine and convert them into useful nutrients, such as short-chain fatty acids (SCFA) and vitamins, which can then be absorbed in the large intestine and used by the animal [3]. Invasion of the gut by pathogens may lead to epithelial cell damage. Thus, the gut may help to defend against this damage by increasing the rate of epithelial renewal and affecting the villus/crypt architecture [4, 5].

It is well known that the abrupt changes in feed composition and feeding conditions at weaning cause a dramatic change in digestive function, which often results in the intestinal malabsorption of nutrients [6, 7]. Therefore, weaning is associated with growth retardation as well as an increase in both morbidity and mortality in neonatal animals [8]. Weaning-induced damage to the gut and the inadequate development of digestive enzymes affect nutrient absorption and result in diarrhea [9]. Weaning with diarrhea could lead to considerable economic loss, and this problem can be ameliorated by nutritional manipulation [10–12]. Antibiotics have been traditionally used to prevent and treat enteric diseases (including diarrhea) induced by weaning stress. However, the continuous use and/or misuse of antibiotics have led to the emergence of drug and antibiotic residues in animal products [13]. Therefore, novel feed additives are needed to replace antibiotics. As potential alternatives to antimicrobial agents, traditional Chinese herbal medicines or their extracts may play significant roles in modulating microbial growth in early weaned animals [14–17].

The Chinese yam, the rhizome of various species of genus *Dioscorea opposita Thunb.* (Dioscoreaceae), has been used to improve gastrointestinal function, alleviate anorexia, and cure diarrhea in traditional Chinese medicine for many years [18]. Starch, a major polysaccharide, is the most abundant carbohydrate in plants of this type (range 20–60%) [19]. However, there have been few investigations on the fermentable and physiological properties of starch present in medicinal plants, especially the Chinese yam. Research on the properties of starch in Chinese yam is very important due to its potential wide applications in the food and biomedical industries.

We hypothesized that dietary supplementation with Chinese Yam polysaccharide (CYP) may enhance gut health in animals. This hypothesis was tested with a rat model by determining in vitro fermentation products, pH value, NH_3_ content, and bacterial community diversity in an anaerobic broth as well as in cecal digesta on days 7 and 21 after the initiation of dietary supplementation with CYP.

## 2. Materials and Methods

### 2.1. Preparation of CYP

The dried and sliced rhizome of Chinese Yam (*Rhizom dioscoreae*, *Dioscorea opposite*, from Henan Province, China) was decocted twice with distilled water (200 g/L, 1 hour each time). Both decoctions were pooled and condensed under low-pressure conditions to give 1 g herb per mL decoction. The condensed solution was purified as follows. (1) The solution was subjected to deproteinization by the dropwise addition of 10% trichloroacetic acid until pH 3.0. (2) After being allowed to age overnight, the supernatant fluid isolated by centrifugation at 3000 g and 4°C for 10 minutes was extracted by precipitation with dehydrated ethanol with a final content of 30% [20]. (3) The precipitates were redissolved in distilled water (200 g/L) and reprecipitated with dehydrated ethanol with a final content of 30%. (4) The upernatant fluid was collected by entrifugation and precipitated with dehydrated ethanol with a final content of 45% in the supernatant fluid. Steps 1 to 3 were repeated, except that the final contents of ethanol were 45%, 60%, and 75%, respectively, and the obtained in each precipitation treatment were purified with the same final contents of ethanol. The polysaccharides (CYP 1, CYP 2, CYP 3, and CYP 4) were obtained by lyophilizing the precipitates collected when the final contents of ethanol were 30%, 45%, 60%, and 75%, respectively. The physical characteristics of the four CYPs are presented in Table 2. The carbohydrate contents (g/kg) in the four final products were 458.5, 562.4, 909.1, and 138.7, respectively, as measured by Vitriol-anthrone [20] using anhydrous glucose as a standard, and contamination of the extracts by protein or nucleic acid was negligible (absorbance at 280 nm and 260 nm was close to zero). The final extracts were used as feed additives in this study.

### 2.2. Experimental Design

This study was carried out in accordance with the Chinese guidelines for animal welfare, and the experimental protocols were approved by the Animal Care Committee, Institute of Subtropical Agriculture, the Chinese Academy of Sciences, Changsha, China [21].

In *Exp. 1*, cecal digesta were collected from 10 weanling SD rats (male : female 1 : 1, average initial body weight of 87 ± 2 g) that had been fed a normal diet (Table 2) and used as the control inoculum, and the four CYPs were used as substrates during 48 hours of fermentation in an anaerobic in vitro system. The fermentation kinetic parameters (including maximum volume and rate of gas production), pH value, NH_3_ content, production and composition of SCFA, and bacterial community diversity in the fermentation broth were determined.

In *Exp. 2*, 40 weanling SD rats with an average initial body weight of 87 ± 3 g were randomly assigned to 4 treatment groups in a randomized complete block design, representing supplementation of a maize and soybean meal-based diet with 0, 0.25, 0.5, or 1.0 g/kg of CYP 3 (Table 1). The results in *Exp. 1* showed that CYP 3 is a good source of carbon and energy, improves bacterial community diversity, and modulates SCFA production in fermentation broth. Therefore, CYP 3 was chosen for use in the in vivo experiment. The basal diet was formulated to meet or exceed the nutrient requirements of growing rats according to laboratory rodent formula feeds (GB 14924.3-2001, China). There were 10 rats (five males and five females) per treatment group, with one rat per cage. Each cage was equipped with a poly-hole feeder and a water nipple to allow ad libitum consumption of feed and water. The temperature and relative humidity were maintained at 28 ± 2°C and 65–75%, respectively, in an air-controlled nursery room. Cecal digesta of four rats per group were collected on days 7 and 21 to determine the pH value, the production and composition of SCFA and bacterial community diversity.

### 2.3. Cecal Inoculums

After a seven-day period of adaptation to the diet, cecal digesta samples were collected and immediately placed in a previously warmed flask (approximately 37°C) that had been filled with CO_2_ in *Exp. 1*. When the warmed flask was received at the laboratory, the interior was flushed with CO_2_. The weight of the cecal digesta was then determined. Before use, and during preparation of the inoculums, continuous bubbling with CO_2_ in a water bath at 37°C maintained anaerobiosis and ensured a constant pH (6.9-7.0). The sample was processed within 1 hour of collection by adding 10 parts of sterile, anaerobic saline and warmed (37°C) nutritive buffer to one part of cecal digesta (v/w) in the insulated bottle, mixing in a blender for 60 seconds, and filtering through a double layer of sterile cheesecloth. The nutritive medium was made from a carbonate-phosphate buffer solution containing (g/L) NaHCO 9.240, Na_2_HPO_3_ *·*12H_2_O 7.125, NaCl 0.470, KC1 0.450, Na_2_SO_4_ 0.100, CaCl_2_ (anhydrous) 0.055, MgCL_2_ (anhydrous) 0.047, and urea 0.400, with added trace elements (10 mL of the following solution (mg/l) per liter of final solution: FeSO_4_ *·*7H_2_O 3680, MnSO_4_ *·*7H_2_O 1900, ZnSO_4_ *·*7H_2_O 440, CoCl_12_ *·*6H_2_O 120, CuSO_4_ *·*5H_2_O 98, Mo_7_ (NH_4_)_6_O_24_ *·*4H_2_O 17.4) [22].

### 2.4. Measurement of Gas Production

In vitro fermentation was carried out in graduated glass syringes (100 mL capacity) [23], with slight modifications to the method used to prepare inoculums. The modifications included the proportion of rat cecal content to carbonate-phosphate buffer solution (1 : 10, w/v), and the content of sample (200 mg of CYPs or glucose in 10 mL of cecal inoculum). The syringes were filled with 10 mL of the resulting mixture as an inoculum. Except for blanks, each oven-dried CYP sample (200 ± 1 mg) was accurately weighed in four replicates into 100-mL glass syringes fitted with plungers and incubated in a shaking water bath at 37°C for 36 hours. At the same time, four syringes that contained only 10 mL of the inoculum or inoculum plus 200 mg glucose were used as negative or positive controls, respectively. In addition, four empty syringes that contained either only the incubation medium or medium plus a different CYP or glucose were incubated as blanks to correct for gas production resulting from the activity of the medium and substrates, respectively. Blanks that contained substrates but no inoculum were also prepared to ensure that selected bacterial species had originated from the cecal digesta and not from the substrate (which was not sterile). Gas production readings were taken after 0.5, 1, 2, 4, 6, 8, 10, 12, 14, 16, 18, 20, 22, 24, 28, 32, and 36 hours of incubation by referring to the moving scale on the plunger of the glass syringes. Fermentation was terminated at 36 hours by instantaneous freezing (dry ice). To describe the dynamics of in vitro gas production over time, the following Gompertz function was used [24]:

(1) $$\text{GP }=\;A\exp\;\left\{-\exp\;\left[1+\frac{Be\left(\text{LAG}-t\right)}{A}\right]\right\},$$

where GP is the cumulative gas production (mL) at time point *t*, *A* is the theoretical maximum gas production (mL), *B* is the maximum rate of gas production (mL/h), LAG (h) is the lag time, *t* is the incubation time (h), and e is the Euler constant (base of the natural logarithm). *A*, *B*, and LAG were calculated using NLREG (Version 5.0) software [25].

### 2.5. Sample Preparation and Analyses

The syringe contents in *Exp. 1* or cecal digesta samples in *Exp. 2* were homogenized by mechanical mixing (vortex) and centrifuged at 1000 g for 10 minutes. The pH of the resulting supernatant fluid was measured by a Delta 320-type pH meter and the NH_3_ contents were determined by a UV-160A UV Spectrophotometer (Shimadzu, Japan) at 550 nm [26].

A mixture of supernatant fluid and 25% metaphosphoric acid solution (4 mL : 1 mL) was prepared for the determination of SCFA (acetic, propionic, butyric, and iso-butyric) by gas chromatography [27]. Briefly, the acidified samples were homogenized and centrifuged at 500 × g for 1 hour. The supernatant portion was filtered through a 0.45 *μ*m polysulfone filter and analyzed using a Hewlett-Packard gas chromatograph with a flame ionization detector and a 1.82 m × 0.2 mm i.d. glass column packed with 10% SP-1200/1% H_3_PO_4_ on 80/100 Chromosorb W AW (HP Inc., USA). A 1-*μ*L sample was used for chromatographic injection, and samples were run in triplicate under the following conditions: column temperature 115°C, injector and detector 200°C, flow rate 15 mL/min and helium carrier 50 psi head pressure. SCFA contents were determined with an HP 5890 Series II Integrator using 2-ethylbutyric acid as an internal standard, and by comparing peaks to known standards (Supelco volatile acids mix, cat no. 4-6975). 

### 2.6. Microbiological Analysis

A molecular approach based on Denaturing Gradient Gel Electrophoresis (DGGE) analysis was used to study the cecal microbial community after in vitro fermentation or dietary supplementation with CYP 3. DNA was extracted from residual samples and used as templates to amplify fragments of the 16S rRNA gene (16S rDNA). PCR products of the V3 regions were analyzed by DGGE. 

#### 2.6.1. Extraction and Purification of DNA from Gut Contents

All of the remaining supernatant fluid from each treatment group in *Exp. 1* was combined into a single sample, kept on ice, and processed further within 2 hours, while the three samples of cecal digesta obtained in *Exp. 2* were analyzed individually (15 wells per Gel). After three washes in saline containing 0.1% Tween 80 with vigorous hand shaking for 5 min per wash, the contents were precipitated by centrifugation (27000 × g, 20 min) at 4°C. The DNA from the precipitate was extracted and purified using a QIAamp DNA Stool Kit (Qiagen, Hilden, Germany) [28], and then stored at −80°C.

#### 2.6.2. PCR Amplification

For PCR-DGGE analysis, each DNA sample was standardized to 20 *μ*g/ml and then amplified using primers that were specific for conserved sequences flanking the variable V3 region of 16S rDNA [29]. Part of the 16S rRNA gene was then amplified by a PCR reaction using a pair of PCR primers that are common to most bacteria, followed by DGGE analysis [30]. The DGGE profiles of microbiota associated with different treatments were compared and analyzed. The 16S rRNA genes were amplified by PCR from genomic DNA of bacteria using eubacterial primers HAD1-GC (5′-CGC CCG GGG CGC GCC CCG GGC GGG GCG GGG GCA CGG GGG GAC TCC TAC GGG AGG CAG CAG T-3′) and HAD2 (5′-GTA TTA CCG CGG CTG CTG GCA C-3′). PCR reaction mixtures were the same as described previously [31]. The amplification program was 94°C for 4 min and 30 cycles of 94°C for 30 s, 56°C for 30 seconds, and 72°C for 2 min, followed by 10 min at 72°C. 

#### 2.6.3. DGGE Analysis

DGGE was performed as described previously using a Bio-Rad D-Code System (Bio-Rad, Calif, USA) [32]. The PCR fragments were separated using 8% polyacrylamide gels with 1.0 × TAE buffer (20 mM Tris-acetate, pH 7.4, 10 mmol/L sodium acetate, 0.5 mmol/L Na_2_EDTA) with 35–60% linear gradients of denaturant (100 % denaturant corresponds to 7 mol/L urea and 40% deionized formamide). The polyacrylamide was diluted from a nondeionized 40% acrylamide/bis stock solution 37.5 : 1 (Bio-Rad, Calif, USA). Gradients were formed using a Bio-Rad Gradient Former Model 385 and gels were polymerized onto a gel support film (FMC, Me, USA). The PCR samples were applied to gels in aliquots of 5 *μ*L per lane. Electrophoresis was performed at 60°C for 16 hours at 100 V. Additionally, bacterial reference ladders, which represented known bacterial strains that included the following species, *Staphylococcus aureus*, *Lactobacillus amylovorus*, *Lactobacillu salivarius*, *Ruminococcus forques*, *Bacillus subtilis*,* E. coli* O157:H7, *Clostridium perfringens, Salmonella typhimurium*, and *Clostridium lituseburense*, listed in order from the top of the gel to the bottom, were loaded to enable the standardization of band migration and gel curvature among different gels [32]. After electrophoresis, gels were silver-stained and scanned using a GS-710 calibrated imaging densitometer (Bio-Rad). Each individual amplicon was then visualized as a distinct band that represented at least one bacterial species on the gel.

#### 2.6.4. DGGE Image Analysis

DGGE images were analyzed using Quantity One v. 4.5.2 software (Bio-Rad, Calif, USA). The software was configured to automatically detect bands on gels. Automatic band detection criteria were identical for all lanes for each gel. When gel imperfections and features were automatically detected as bands by the software, these false bands were manually removed and not included in subsequent numerical analyses. Anomalous staining residues (spotting and peppering) were removed from digital images of gels as necessary. DGGE profiles were also compared using Sorenson's index, a pairwise similarity coefficient *C*_*s*_, which was determined as *C*_*s*_ = [2*j*/(*a* + *b*)] × 100, where *a* is the number of DGGE bands in lane 1, *b* is the number of DGGE bands in lane 2, and *j* is the number of common DGGE bands [33]. Dendrograms were constructed using the unweighted pair group method with an arithmetic mean.

### 2.7. Statistical Analysis

Data are expressed as the mean ± S.D. Results were statistically analyzed using one-way ANOVA (SAS Institute, Cary, NC). Duncan's multiple range test was used to compare differences among the treatment groups. A *P*-value of less than 0.05 was considered statistically significant.

## 3. Results

### 3.1. In Vitro Gas Production (GP)

In vitro gas production parameters of CYPs and glucose are given in Table 3. GP and *B* in the CYP 3-supplemented group were higher (*P* < .05) than those in the other CYP-supplemented groups and the non-supplemented group, but did not differ (*P* > .05) from those in the glucose-supplemented group. With the exception of the control group (6.17 mL/h and 0.37 mL/h), the lowest values for GP (27.72 mL/h) and *B* (1.59 mL/h) were observed with CYP 2 and CYP 1, respectively. The LAG values in the CYP 2-, CYP 3-, and CYP 4-supplemented groups were lower (*P* < .05) than those in the other groups. The greatest LAG value (1.55 h) was observed in the control group, which was higher (*P* < .05) than those in the CYP 1-supplemented group (0.93) and glucose-supplemented group (0.69). There was no significant difference (*P* > .05) in GP and *B* between the CYP 3- and glucose-supplemented groups.

**Figure 1:**
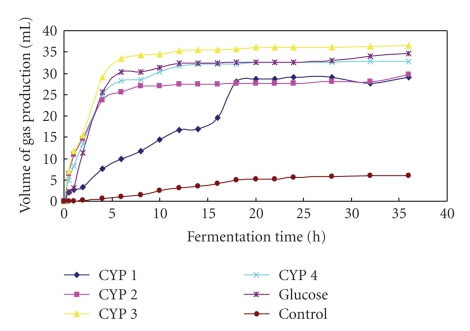
Time-course of gas production by intestinal microbes in response to in vitro supplementation with Chinese Yam polysaccharide (CYP) or glucose (*n* = 4).

The digestion curves for the four CYPs and glucose are shown in Figure 1. The general shapes of the curves in the CYP 2-, CYP 3-, CYP 4-, and glucose-supplemented groups, but not the control group, were similar, in that they showed an apparent lag, a rapidly increasing rate of digestion, and an asymptotic approach to maximum digestion. Supplementation with CYP 3 was associated with the highest rate of digestion, while CYP 2 yielded the lowest rate of digestion compared to the glucose and CYP 4-supplemented groups.

### 3.2. pH Values and NH_3_ Contents in Fermentation Broth and Cecal Digesta

The pH values and NH_3_ contents in fermentation broth are summarized in Table 4. The pH values in the CYP 2-, CYP 3-, CYP 4-, and glucose-supplemented groups were lower (*P* < .05) than those in the other two groups, and there were no differences (*P* > .05) among the 4 supplementation groups. Supplementation with CYP 2 and CYP 3 decreased (*P* < .05) the NH_3_ contents in the fermentation broth compared to the CYP 1- and nonsupplemented groups, but increased (*P* < .05) the NH_3_ contents compared to the glucose-supplemented group. 

Dietary supplementation with 0.25 or 0.5 g/kg CYP 3 decreased the pH value of cecal digesta on day 7 after the initiation of supplementation compared to the 1 g/kg CYP 3-supplemented group (*P* < .05), while there were no differences (*P* > .05) on day 21 among the four groups. The NH_3_ contents in cecal digesta did not differ (*P* > .05) among the CYP-supplemented groups and non-supplemented group on days 7 and 21 after the initiation of supplementation (data not shown).

### 3.3. Production and Composition of SCFA in Fermentation Broth and Cecal Digesta 

While supplementation with CYP 3 increased (*P* < .05) the contents of acetate, propionate, and butyrate compared to those in the CYP 1-, CYP 2-, and non-supplemented groups, there were no differences (*P* > .05) from the CYP 4-, and glucose-supplemented groups, with the exception of butyrate (Table 5). The contents of isobutyrate in the CYP 2-, CYP 3-, CYP 4-, and glucose-supplemented groups were lower (*P* < .05) than those in the non-supplemented group, but greater (*P* < .05) than those in the CYP 1-supplemented group. On day 7 of the feeding trial, the contents of acetate and butyrate in the cecal digesta of rats fed 0.25 and 0.5 g/kg of CYP 3 were higher (*P* < .05) than those in the other two groups. The contents of propionic acid in rats fed 0.25 g/kg of CYP 3 were higher than those in the other three groups. Dietary supplementation with 0.25 or 0.5 g/kg of CYP 3 increased (*P* < .05) the contents of acetate at the end of the feeding trial in comparison with the other two groups (Table 6). The contents of isobutyrate in cecal digesta did not differ (*P* > .05) among the CYP-supplemented groups and non-supplemented group on days 7 and 21 after the initiation of supplementation (data not shown).

### 3.4. Correlations among pH Values and Contents of N*H*_3_ and SCFA after in Vitro Fermentation

The correlations between the pH values and the contents of NH_3_ and SCFA after in vitro fermentation are presented in Table 7. The pH value and NH_3_ content were negatively correlated with the contents of acetate (−0.89 or −0.86, *P* < .05), propionate (−0.96, *P* < .01; or −0.86, *P* < .05), and butyrate (−0.96, *P* < .01; or, −0.83, *P* < .05), while there were no significant correlations (*P* > .05) between the isobutyrate content and the pH value or NH_3_ content. Positive correlations were observed between the contents of acetate and those of propionate (0.92, *P* < .01) and butyrate (0.90, *P* < .05), as well as between the contents of propionate and butyrate (0.95, *P* < .01), and the pH value and NH_3_ content (0.94, *P* < .01).

### 3.5. Bacterial Community Diversity in Fermentation Broth under the Presence of CYPs or Glucose 

**Figure 2:**
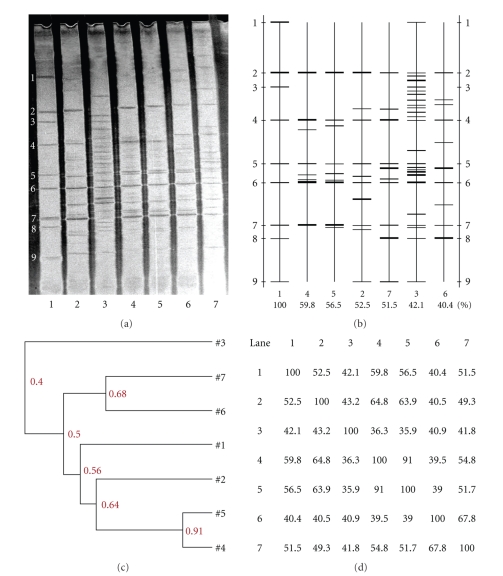
(a) DGGE profiles of V_3_ amplicons in the fermentation broth of cecal microbiota from weaned rats in the presence of glucose (2), culture solution (3), CYP 4 (4), CYP 3 (5), CYP 2 (6), and CYP 1 (7) for 48 hours. The standard strains (1) listed in order from the top of the gel to the bottom were *Staphylococcus aureus, Lactobacillus amylovorus*, *Lactobacillus salivarius*, *Ruminococcus forques*, *Bacillus subtilis, E.coli* O157: H7, *Clostridium perfringens*, *Salmonella typhimurium* and *Clostridium lituseburens,* respectively. (b) Sketch map of the DGGE profiles. (c) and (d) Similarity indices of the DGGE profiles. All of the four samples from each treatment group were pooled into a single sample.

The DGGE profiles of V_3_ amplicons obtained from the in vitro fermentation broth of cecal microbiota in rats in the presence of CYPs or glucose are shown in Figure 2(a). The changes in the number and intensity of bands in DGGE lanes reflected the complexity and diversity of the bacterial populations in the different treatment groups (Figure 2(b)). The effects of CYPs and glucose on the richness of intestinal bacterial populations in rats are summarized in Tables 8 and 9. The DGGE profiles (Figure 2(a)) and aketch map (Figure 2(b)) indicated that there were 10, 25, 8, 9, 10, and 10 bands from the fermentation broth of cecal microbiota with glucose (2), culture solution (3), CYP 4 (4), CYP 3 (5), CYP 2 (6), and CYP 1 (7), respectively (Table 8). The change in the intensity of bands in DGGE lanes showed that the presence of CYP 3, CYP 4, or glucose in the fermentation broth increased the abundance of bacterial species with a distance of migration similar to the DNA references, including *Lactobacillus amylovorus*, and CYPs or glucose also increased *Ruminococcus forques* (Table 9). In contrast, CYPs or glucose decreased *Staphylococcus aureus* and *Lactobacillus salivarius*. Interestingly, CYP 1 and CYP 2 increased *Salmonella typhimurium*, but decreased *E. coli* O157:H7 (Table 9). The presence of CYP promoted the development of beneficial gut microbiota and suppressed bacterial pathogens, based on the DGGE profiles (Figures 2(c) and 2(d)). The similarity indices between the CYP 3- and CYP 4-supplemented groups (91.0%), or the CYP 1- and CYP 2-supplemented groups (67.8%), were fairly high.

### 3.6. Microbiota Diversity in Cecal Samples of Rats Supplemented with Various Doses of CYP 3 

**Figure 3:**
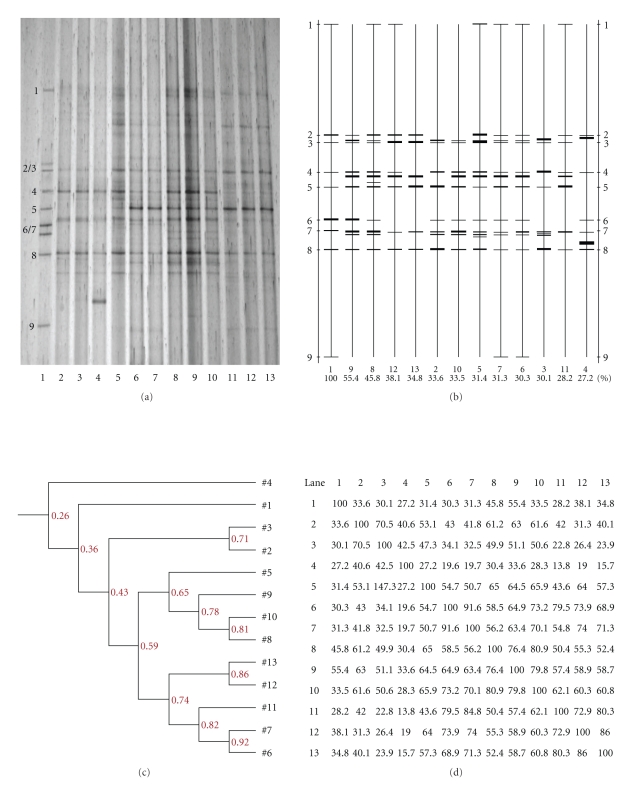
(a) DGGE profiles of V_3_ amplicons in the cecal microbiota of weaned rats that were fed diets supplemented with 0 (2 to 4), 0.25 (5 to 7), 0.5 (8 to 10), and 1.0 (11 to 13) g/kg CYP 3 on day 7 after the initiation of supplementation. The standard strains (1) listed in order from the top of the gel to the bottom were *Staphylococcus aureus*, *Lactobacillus amylovorus*, *Lactobacillus salivarius*, *Ruminococcus forques*, *Bacillus subtilis*, *E. coli* O157 : H7, *Clostridium perfringens*, *Salmonella typhimurium* and *Clostridium ituseburense,* respectively. (b) Sketch map of the DGGE profiles. (c) and (d) Similarity indices of the DGGE profiles.

**Figure 4:**
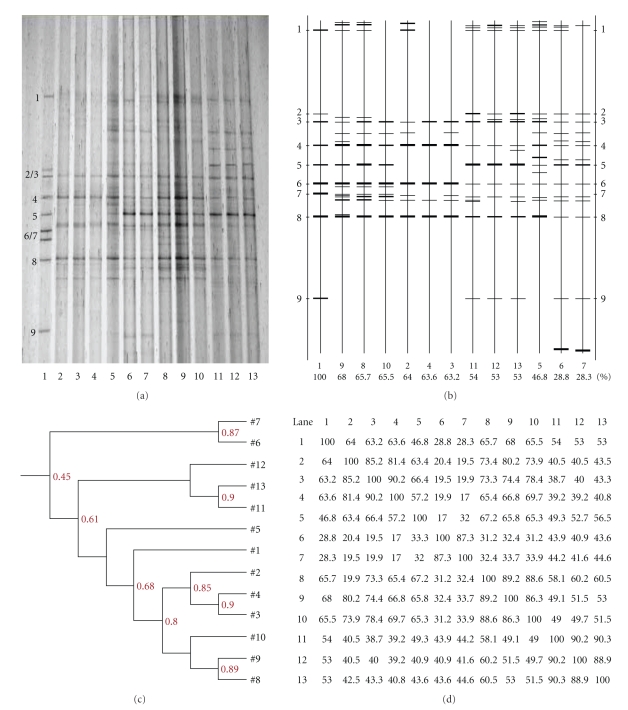
(a) DGGE profiles of V_3_ amplicons in cecal microbiota of rats fed diets supplemented with 0 (2 to 4), 0.25 (5 to 7), 0.5 (8 to 10), and 1.0 (11 to 13) g/kg CYP 3 on day 21 after the initiation of supplementation. The standard strains (1) listed in order from the top of the gel to the bottom were *Staphylococcus aureus*, *Lactobacillus amylovorus*, *Lactobacillus salivarius*, *Ruminococcus forques*, *Bacillus subtilis*, *E. coli* O157:H7, *Clostridium perfringens*, *Salmonella typhimurium*, and *Clostridium lituseburense*, respectively. (b) Sketch map of the DGGE profiles. (c) and (d) Similarity indices of the DGGE profiles.

Figures 3(a) and 4(a)  show DGGE profiles of V_3_ amplicons obtained from cecal microbiota in rats that had been supplemented with various doses of CYP 3 on days 7 and 21 after the initiation of supplementation, respectively. The changes in number and intensity of bands in DGGE lanes reflected the complexity and diversity of the bacterial population in the different treatment groups Figures 3(b) and 4(b)). The effects of various doses of CYP 3 on the richness of intestinal bacterial populations in rats are summarized in Tables 10 and 11.

The DGGE profiles (Figure 3(a)) and a ketch map (Figure 3(b)) indicated that there were 8, 8, 7; 10, 1, 1; 1, 10, 9; 7, 7, 6 bands from the cecal microbiota of rats supplemented with 0 (2 to 4), 0.25 (5 to 7), 0.5 (8 to 10), and 1.0 (11 to 13) g/kg CYP 3 on day 7 after the initiation of supplementation, respectively (Table 10). The change in band intensity in DGGE lanes showed that dietary supplementation with CYP 3 increased abundance of bacterial species with a similar to the DNA references, including *Lactobacillus amylovorus*, but eased *Salmonella typhimurium*. Additionally, 1.0 g/kg of CYP 3 increased *Lactobacillus salivarius* and *Bacillus subtilis*, but decreased *Ruminococcus forques* and *E. coli* O157:H7 on day 7 after the initiation of supplementation (Table 11). The presence of CYP promoted the development of beneficial gut microbiota, and suppressed bacterial pathogens (Figures 3(c) and 3(d)). The similarity indices among Lanes 5, 6, and 7 (59%); 8, 9, and 10 (78%), and 11, 12, and 13 (74%) were fairly high.

The DGGE profiles (Figure 4(a)) and a sketch map (Figure 4(b)) indicated that there were 9, 7, 5; 15, 1, 14; 14, 15, 10; 12, 12, 14 bands from the cecal microbiota of rats supplemented with 0 (2 to 4), 0.25 (5 to 7), 0.5 (8 to 10), and 1.0 (11 to 13) g/kg CYP 3 on day 21 after the initiation of supplementation, respectively (Table 10). The change in band intensity in DGGE lanes on day 21 after the initiation of supplementation showed that dietary supplementation with CYP 3 increased the abundance of bacterial species with a distance of migration similar to the DNA references, including *Bacillus subtilis*. Similarly, 1.0 g/kg of CYP 3 increased *Lactobacillus amylovorus* and *Clostridium* but decreased *Ruminococcus forques*, *E. coli* O157: H7, and *Salmonella typhimurium* (Table 11). Figures 4(c) and 4(d)  show the similarity indices of the DGGE profiles. The similarity indices among (1) Lanes 2, 3, and 4 (85%), (2) 8, 9, and 10 (85%), and (3) 11, 12, and 13 (85%) were fairly high.

## 4. Discussion

The PCR-DGGE technique can be combined with in vitro fermentation as a preliminary test to study changes in microbial populations when gut microbiota are exposed to a single source of fermentable carbohydrate as a source of energy [22, 32]. Observed shifts can be used to indicate which microbial species would most likely be favored in the gut if that carbohydrate-based ingredient were to be included in the animal diet as a prebiotic. In the present work, a PCR-DGGE approach was used to examine bacterial changes in the cecal digesta of rats following an in vitro fermentation and feeding trial by examining the microbial ecosystem after the in vitro fermentation of CYP and dietary supplementation with CYP. The PCR-DGGE analysis showed that CYP 3 promoted the population of beneficial gut microbiota with the same migration distance as *Lactobacillus amylovorus* and *Ruminococcus forques*, but suppressed bacterial pathogens that comigrated with *Staphylococcus aureus, Salmonella typhimurium,* and *Clostridium lituseburense*. Therefore, CYP 3 was chosen for use in an in vivo study. 

Various techniques, including PCR-DGGE analysis, have shown that some nondigestible fermentable food ingredients can selectively stimulate the growth and/or activity of one or a limited number of bacteria of the gut microflora [16, 30, 34], though usually the main emphasis has been placed on *Bifidobacteria* and *Lactobacilli* [31, 35]. In this study, the DGGE profiles in the feeding trial indicated that the gut microflora in CYP 3-supplemented rats exhibited greater diversity than those in non-supplemented rats. Consistent with the earlier finding, CYP 3 enriched the density of gut microbiota with the same migration distance as *Lactobacillus amylovorus* and *Bacillus subtilis*, but suppressed bacterial pathogens that co-migrated with *Salmonella typhimurium* in rat cecum.

An increase in the amount of some beneficial gut microbiota can generate high SCFA and low ammonia contents compared to other feed ingredients [36, 37]. In addition, since it is readily fermented by these populations, it has been considered to be a useful ingredient to test in weaner diets, so that it might smooth the transition of the microbial population as the neonatal animal experienced a change from milk to a solid diet [3]. In our in vitro fermentation study, the contents of acetate, propionate, and butyrate were increased compared to supplementation with CYP 3 or CYP 4 while the isobutyrate contents in the four CYPs groups were decreased (*P* < .05) compared to that in the control group. The fermentation process provides metabolic end products for the colon (mainly acetate, propionate, and butyrate) and supplies energy for the growth or maintenance of intestinal microflora. Butyrate appears to be an important physiologically significant source since it is the major fuel for colonic epithelial cells even when competing substrates, such as glucose and glutamine, are available [38]. The major gases produced in the fermentation process include hydrogen, carbon dioxide, and methane [36]. The maximum volume and rate of gas production in the CYP 3-supplemented group in this study were higher (*P* < .05) than those in the control group, while the pH values and NH_3_ contents in the CYP 3-supplemented group were lower (*P* < .05) than those in the control group. These findings suggest that CYP 3, as a phytochemical dietary additive, is a good source of carbon and energy for the intestines and may improve bacterial community diversity and modulate SCFA production. Organic acids exhibit potential antimicrobial activity, since their undissociated forms pass freely through the cellular membrane of pathogenic microflora [39]. 

In vitro gas production parameters usually reflect the characteristics of the fermentation process. Moreover, some of the LAG values in the present study were negative, such as in the CYP 2- (−0.28), CYP 3- (−0.15), and CYP 4 (−0.11) supplemented groups. Krishnamoorthy et al. also observed negative LAG values for in vitro gas curves of oat, rye, and hay standards incubated for 96 hours. They assumed that the negative lag-time values are a consequence of rapid gas production in the early stages of fermentation, which may have been caused by the characteristics of the fermentable substrate [40]. The products of microbial metabolism may regulate the digestion and absorption of dietary nutrients, including amino acids and minerals [41–43], thereby affecting the growth and health of animals.

In summary, we demonstrated that CYP 3, one of four fractions of the Chinese Yam polysaccharide, had the strongest bioactivity. CYP 3 enriched beneficial gut microbiota, suppressed bacterial pathogens in the rat cecum, and increased SCFA production. These findings suggest that CYP 3 may serve as a useful dietary supplement for improving gut heath in weanling rats.

## Figures and Tables

**Table 1:** Dietary ingredients and nutrient levels of the basal diet (g/kg, as-fed basis).

Dietary ingredients		Nutrient levels^(a)^	
Corn (CP, 8.7%)	380	Digestible energy (MJ/kg)	14.2
Soybean meal (CP, 43.0%)	370	Crude protein	243
Wheat bran (15.7%)	130	Ether extract	65
Fish meal (62.5%)	50	Crude fiber	36
Soybean oil	30	Crude ash	76
NaCl	10	Total Ca	11.6
Mineral mixture^(1)^	15	Total P	8
Vitamin mixture^(2)^	15		

^(a)^Analyzed values for all dietary components.^(1)^Provided the following amount per kilogram of diet: Cu: 13 mg; Fe: 270 mg; Mn: 64 mg; Zn: 70 mg; I: 0.8 mg; Se: 0.27 mg; Co: 0.6 mg; ^(2)^Provided the following amount per kilogram of diet: vitamin A: 14000 IU; vitamin D3: 1500 IU; vitamin E: 120 IU; vitamin K_3_: 5 mg; thiamine: 13 mg; riboflavin: 12 mg; pyridoxine: 12 mg; vitamin 12: 0.022 mg; biotin: 0.2 mg; d-pantothenic acid: 24 mg; folic acid: 6 mg; niacin: 60 mg; choline: 1250 mg.

**Table 2:** Physical characteristics and net content of the four fractions of Chinese Yam polysaccharide (CYP).

CYP	Physical characteristics of CYP		Polysaccharide content (g/kg)
	Wet	Dry	
CYP 1	White, low viscosity	White, loose	458.5
CYP 2	Light gray, moderately iscous	Light gray, firm	562.4
CYP 3	Gray, highly iscous	Gray, firm	909.1
CYP 4	White, somewhat viscous	White, slightly firm	138.7

**Table 3:** In vitro gas production parameters with Chinese Yam polysaccharide (CYP) and glucose (*n* = 4).

Treatment	Maximum volume of gas production (GP, mL)	Rate of gasproduction (*B*, mL/h)	Lag time of gasproduction (LAG, h)
CYP 1	30.82 ± 0.47^(bc)^	1.59 ± 0.09^(c)^	0.93 ± 0.50^(b)^
CYP 2	27.72 ± 3.09^(c)^	7.04 ± 0.99^(b)^	(−0.28) ± 0.15^(c)^
CYP 3	35.72 ± 0.88^(a)^	7.99 ± 0.52^(a)^	(−0.15) ± 0.18^(c)^
CYP 4	32.06 ± 2.96^(b)^	6.44 ± 0.36^(b)^	(−0.11) ± 0.07^(c)^
Glucose	32.65 ± 2.17^(ab)^	8.49 ± 0.54^(a)^	0.69 ± 0.10^(b)^
Control	6.17 ± 0.08^(d)^	0.37 ± 0.01^(d)^	1.55 ± 0.05^(a)^

^(a−d)^Mean ± S.D. values with different superscripts within a column are significantly different (*P* < .05).

**Table 4:** pH values and NH_3_ contents in the fermentation broth (*n* = 4).

Treatment	pH value	NH_3_ content (mg/dL)
CYP 1	6.14 ± 0.18^(b)^	17.54 ± 2.32^(b)^
CYP 2	5.11 ± 0.09^(c)^	12.77 ± 2.63^(c)^
CYP 3	4.88 ± 0.12^(c)^	12.44 ± 1.42^(c)^
CYP 4	5.05 ± 0.11^(c)^	14.82 ± 1.64^(bc)^
Glucose	4.92 ± 0.33^(c)^	8.33 ± 1.19^(d)^
Control	7.28 ± 0.08^(a)^	24.43 ± 0.69^(a)^

^(a−d)^Mean ± S.D. values with different superscripts within a column are significantly different (*P* < .05).

**Table 5:** Contents of short-chain fatty acids in fermentation broth (*μ*mol/100 mL; *n * = 4).

Treatment	Acetate	Propionate	Isobutyrate	Butyrate
CYP 1	478.8 ± 23.0^(d)^	363.5 ± 84.2^(c)^	80.1 ± 6.5^(d)^	188.8 ± 14.9^(c)^
CYP 2	566.7 ± 49.7^(c)^	790.0 ± 9.3^(b)^	105.1 ± 17.4^(bc)^	218.8 ± 19.8^(b)^
CYP 3	803.3 ± 32.6^(ab)^	903.7 ± 90.8^(a)^	104.4 ± 6.4^(bc)^	258.9 ± 12.4^(a)^
CYP 4	745.9 ± 39.9^(b)^	945.7 ± 45.9^(a)^	111.9 ± 1.6^(b)^	258.6 ± 9.6^(a)^
Glucose	823.8 ± 86.5^(a)^	875.6 ± 10.5^(ab)^	89.8 ± 8.6^(bcd)^	233.2 ± 43.6^(b)^
Control	381.8 ± 17.9^(e)^	189.2 ± 7.7^(c)^	135.5 ± 7.2^(a)^	128.2 ± 5.0^(d)^

^(a−e)^Mean ± S.D. values with different superscripts within a column are significantly different (*P* < .05).

**Table 6:** Contents of short-chain fatty acids and pH value in cecum in weaned rats (*μ*mol/g, *n* = 4).

	Acetate	Propionate	Butyrate	pH value
Day 7 after the initiation of supplementation				

0	3.78 ± 0.24^(b)^	5.20 ± 0.26^(b)^	2.35 ± 0.24^(b)^	7.09 ± 0.48^(ab)^
0.25 g/kg CYP 3	5.50 ± 0.37^(a)^	6.35 ± 0.66^(a)^	2.93 ± 0.28^(a)^	6.61 ± 0.48^(b)^
0.5 g/kg CYP 3	5.15 ± 0.26^(a)^	5.43 ± 0.35^(b)^	2.88 ± 0.31^(a)^	6.50 ± 0.26^(b)^
1.0 g/kg CYP 3	3.95 ± 0.47^(b)^	4.98 ± 0.85^(b)^	2.28 ± 0.30^(b)^	7.44 ± 0.20^(a)^

Day 21 after the initiation of supplementation				

0	4.60 ± 0.24^(c)^	6.10 ± 0.37	3.20 ± 0.26^(a)^	6.96 ± 0.11
0.25 g/kg CYP 3	5.78 ± 0.34^(a)^	6.75 ± 0.75	3.50 ± 0.62^(a)^	7.19 ± 0.14
0.5 g/kg CYP 3	5.30 ± 0.22^(b)^	6.58 ± 0.38	3.03 ± 0.22^(ab)^	7.57 ± 0.33
1.0 g/kg CYP 3	4.58 ± 0.30^(c)^	6.05 ± 0.19	2.58 ± 0.21^(b)^	7.53 ± 0.35

^(a−c)^Mean ± S.D. values with different superscripts within a column are significantly different (*P* < .05).

**Table 7:** Correlations among pH value and contents of NH_3_ and short-chain fatty acids after in vitro fermentation.

* *	Propionate	Isobutyrate	Butyrate	pH value	NH_3_
Acetate	0.92**	NS	0.90*	−0.89*	−0.86*
Propionate		NS	0.95**	−0.96**	−0.86*
Isobutyrate			NS	NS	NS
Butyrate				−0.96**	−0.83*
pH value					0.94**

NS: *P* > .05; *P < .05; **P < .01.

**Table 8:** Effects of Chinese Yam polysaccharide (CYP) and glucose on the complexity of the intestinal bacterial population in fermentation broth.

Culture solution	Glucose	CYP 4	CYP 3	CYP 2	CYP 1
25	10	8	9	10	10

The data indicate the numbers of bands in DGGE lanes, which reflects the complexity of the bacterial population in the different treatment groups, as also indicated in Figures 2(a) and 2(b). The four samples from each treatment group in *Exp. 1* were pooled into a single sample.

**Table 9:** Effects of Chinese Yam polysaccharide (CYP) on he ntestinal bacterial population in fermentation broth.

DNA bands co-migrating with	Culture solution	Glucose	CYP 4	CYP 3	CYP 2	CYP 1
*Staphylococcus aureus *	±	−	−	−	−	−
*Lactobacillus amylovorus *	±	+	+	+	±	±
*Lactobacillus salivarius *	+	−	−	−	−	−
*Ruminococcus forques *	±	+	++	+	+	++
*Bacillus subtilis *	+	+	+	±	−	+
*E. coli* O157:H7	++	+	++	++	+	+
*Clostridium perfringens *	±	±	++	++	±	±
*Salmonella typhimurium *	+	−	−	−	++	++
*Clostridium lituseburens *	+	+	−	−	−	++

“−” means the bacteria is absent in this part of the intestine; “±,” “+,” and “++” mean that the bacteria is present in this part of intestine and has a lower, equal or higher intensity compared to the standard strain, as also indicated in Figures 2(a) and 2(b). The four samples from each treatment group in *Exp. 1* were pooled into a single sample.

**Table 10:** Effects of Chinese Yam polysaccharide (CYP) 3 on the complexity of the intestinal bacterial population from cecal digesta in weaned rats (*n* = 3). The data indicate the numbers of bands in DGGE lanes, which reflects the complexity of the bacterial population in the different treatment groups, as also indicated in Figures 3(a) or 4(a) and 3(b) or 4(b), respectively.

	Dose of CYP 3 (g/kg)											
	0			0.25			0.5			1.0		
Day 7 after the initiation of supplementation	No. 1	No. 2	No. 3	No. 1	No. 2	No. 3	No. 1	No. 2	No. 3	No. 1	No. 2	No. 3
	8	8	7	10	11	11	11	10	9	7	7	6

	Dose of CYP 3 (g/kg)											
	0			0.25			0.5			1.0		

Day 21 after the initiation of supplementation	No. 1	No. 2	No. 3	No. 1	No. 2	No. 3	No. 1	No. 2	No. 3	No. 1	No. 2	No. 3
	9	7	5	15	15	14	14	15	10	12	12	14

**Table 11:** Effects of Chinese Yam polysaccharide (CYP) 3 on the intestinal bacterial population from cecal digesta in weaned rats (*n* = 3).

DNA bands co-migrating with	Dose of CYP 3 (g/kg)											
	0			0.25			0.5			1.0		
Day 7 after the initiation of supplementation												
	No. 1	No. 2	No. 3	No. 1	No. 2	No. 3	No. 1	No. 2	No. 3	No. 1	No. 2	No. 3

*Staphylococcus aureus*	−	−	−	++	+	+	+	−	−	+	+	−
*Lactobacillus amylovorus*	−	−	±	++	±	±	+	±	−	±	+	+
*Lactobacillus salivarius*	+	+	−	++	+	+	+	+	+	+	++	++
*Ruminococcus forques*	++	++	+	++	+	+	++	++	++	+	+	−
*Bacillus subtilis*	++	+	−	+	++	++	++	++	++	++	++	++
*E. coli* O157:H7	±	−	±	−	±	±	±	+	±	−	−	−
*Clostridium perfringens*	+	+	±	+	+	±	++	++	++	+	±	+
*Salmonella typhimurium*	++	++	+	++	±	±	+	+	+	±	+	+
*Clostridium lituseburense *	−	−	−	−	+	+	−	−	−	−	−	−

Day 21 after the initiation of supplementation												
	No. 1	No. 2	No. 3	No. 1	No. 2	No. 3	No. 1	No. 2	No. 3	No. 1	No. 2	No. 3

*Staphylococcus aureus*	+	−	−	±	±	−	±	±	−	±	±	±
*Lactobacillus amylovorus*	−	−	−	+	+	+	−	−	−	++	+	++
*Lactobacillus salivarius*	±	+	+	+	±	±	+	±	+	+	+	+
*Ruminococcus forques*	++	++	++	+	±	±	++	++	+	±	±	±
*Bacillus subtilis*	−	−	−	±	+	+	++	+	+	++	++	++
*E. coli* O157:H7	+	+	+	±	±	±	+	+	+	±	±	±
*Clostridium perfringens*	±	±	−	±	±	±	−	±	±	±	±	±
*Salmonella typhimurium*	+	+	+	+	±	±	+	+	+	±	±	±
*Clostridium lituseburense *	−	−	−	−	±	±	−	−	−	±	±	±

“−” means the bacteria is absent in this part of the intestine; “±,” “+,” and “++” mean that the bacteria is present in this part of intestine and has a lower, equal, or higher intensity compared to the standard strain, as also indicated in Figures 2(b) and 3(b), respectively.
